# Defining Contemplative Science: The Metacognitive Self-Regulatory Capacity of the Mind, Context of Meditation Practice and Modes of Existential Awareness

**DOI:** 10.3389/fpsyg.2016.01788

**Published:** 2016-11-17

**Authors:** Dusana Dorjee

**Affiliations:** School of Psychology, Bangor UniversityBangor, UK

**Keywords:** contemplative science, meditation, neuroscience, mindfulness, mechanisms, definition, self-regulation, awareness

## Abstract

The term ‘contemplative’ is now frequently used in the fast growing field of meditation research. Yet, there is no consensus regarding the definition of contemplative science. Meditation studies commonly imply that contemplative practices such as mindfulness or compassion are the subject of contemplative science. Such approach, arguably, contributes to terminological confusions in the field, is not conducive to the development of an overarching theory in contemplative science, and overshadows its unique methodological features. This paper outlines an alternative approach to defining contemplative science which aims to focus the research on the core capacities, processes and states of the mind modified by contemplative practices. It is proposed that contemplative science is an interdisciplinary study of the metacognitive self-regulatory capacity (MSRC) of the mind and associated modes of existential awareness (MEA) modulated by motivational/intentional and contextual factors of contemplative practices. The MSRC is a natural propensity of the mind which enables introspective awareness of mental processes and behavior, and is a necessary pre-requisite for effective self-regulation supporting well-being. Depending on the motivational/intentional and contextual factors of meditation practice, changes in the metacognitive self-regulatory processes enable shifts in MEA which determine our sense of self and reality. It is hypothesized that changes in conceptual processing are essential mediators between the MSRC, motivational/intentional factors, context of meditation practice, and the modulations in MEA. Meditation training fosters and fine-tunes the MSRC of the mind and supports development of motivational/intentional factors with the ultimate aim of facilitating increasingly advanced MEA. Implications of the proposed framework for definitions of mindfulness and for future systematic research across contemplative traditions and practices are discussed. It is suggested that the proposed definition of contemplative science may reduce terminological challenges in the field and make it more inclusive of varied contemplative practices. Importantly, this approach may encourage development of a more comprehensive contemplative science theory recognizing the essential importance of first- and second-person methods to its inquiry, thus uniquely contributing to our understanding of the mind.

## Introduction

Research investigating meditation-based techniques has over the last two decades greatly expanded and grown – not only in the simple terms of study numbers, but also considering the variety of meditation techniques investigated, quality of research, its applications and understanding of possible underlying mechanisms. While research on mindfulness dominates the field, increasing numbers of studies investigate compassion (e.g., Pace et al., 2009; Desbordes et al., 2012), visualization-based meditation practices (e.g., Kozhevnikov et al., 2009), equanimity (e.g., Desbordes et al., 2014), etc.; and there are also repeated calls for more studies on non-Buddhist meditation (e.g., Dahl et al., 2015). In meditation-based intervention research, randomized studies with active controls are becoming the golden standard (e.g., Williams et al., 2014; Malinowski et al., 2015), and more rigorous meta-analyses are providing stronger cumulative evidence (e.g., Warren et al., 2016). Understanding of the possible mechanisms leading to the well-being enhancing effects of meditation has also improved, particularly due to theoretical advances (e.g., Shapiro et al., 2006; Garland et al., 2015) and neurocognitive research (e.g., Tang et al., 2015). All these developments suggest that the field is moving away from initial questions investigating *whether* meditation can produce measurable changes in health and well-being, and toward increasing research rigor of studies with closer focus on *how* meditation techniques modify the mind and brain.

This progression from a nascent to a more mature stage of meditation research is also starting to highlight some persistent challenges specific to the field such as terminological unclarities (e.g., Rosch, 2007; Nash et al., 2013; Lutz et al., 2015), particular methodological issues (Davidson and Kaszniak, 2015), and a lack of an overarching theory. Many of the terminological problems relate to differences in meaning of constructs within the traditional Buddhist context and current research – the term ‘mindfulness’ is a prominent example of this (Williams and Kabat-Zinn, 2013), together with constructs such as ‘direct perception,’ ‘insight,’ etc. (Rosch, 2007; Dorjee, 2010). Such confusions have tangible implications for research; for example, if definitions of mindfulness differ across traditions is it appropriate to combine studies on secular mindfulness, studies with Zen practitioners, Insight meditators, and Tibetan Buddhist practitioners in reviewing the impact of mindfulness on neurocognitive mechanisms (e.g., Tang et al., 2015)?

The terminological challenges are likely to increase further as the research on meditation becomes more inclusive of a broader variety of contemplative^1^ practices: ranging from contemplative inquiry and focused meditation on sensory experience, through mantra recitation and visualization, to movement and energy-based practices. And even within the same type of meditation practice such as focused meditation (Lutz et al., 2008), we are likely to find large differences according to the context of the practice (e.g., spiritual/religious or secular), object (breath, sacred images, verbal contemplation, etc.), and format of the contemplative practice (different types of retreat, formal and informal practice). All these variations could contribute to differential modulations of psychological and neurocognitive mechanisms modified by meditation, but are easy to overlook if we use broad categories of meditation types as the subject of research in contemplative science. This highlights the need for greater distinctions between practices, their processes and outcomes, combined with a requirement to integrate the similarities and differences between findings into a strong discipline-specific theoretical framework. Accordingly, increasing numbers of theoretical studies are trying to address definitional (e.g., Vago and Silbersweig, 2012) and classification issues in meditation research (Lutz et al., 2008; Josipovic, 2010; Travis and Shear, 2010; Nash et al., 2013; Dahl et al., 2015).

Aside from terminological and classification challenges, there are methodological issues specific to the field of contemplative science. The main one relates to the introspective dimension of contemplative practices and associated difficulty in capturing the first-person and second-person data. The first-person information refers to the phenomenological ‘what it is like’ aspects of practitioner’s meditation experience only the practitioner has access to. The second-person data is usually provided by an experienced meditation practitioner, often a teacher, who can report in an informed way on another’s, usually a student’s, phenomenological aspects of practice experience. These types of data also need to be meaningfully linked with third person data from behavioral, physiological, and imaging measurements (Varela, 1996; Davidson and Kaszniak, 2015; Lutz et al., 2015). Another methodological challenge is associated with the context of contemplative practices (their secular, spiritual, and religious aspects) which is increasingly acknowledged in theory as an important factor modulating the outcomes and mechanisms (e.g., Shapiro et al., 2006; Dorjee, 2010), but is very rarely considered and assessed in empirical research. As a result, most studies take a limited view of meditation focused on a particular practice taken out of a complex contemplative system, without a more systemic perspective of the role the practice plays in a person’s life-long trajectory of well-being and purpose.

Interestingly, all these terminological, theoretical, and methodological challenges seem to point to the same fundamental limitation of the field which remains unaddressed – the lack of definitional clarity about contemplative science and its subject. Most current studies of meditation implicitly assume that the contemplative practices themselves (often without distinction equated with processes and outcomes they produce) are the subject of contemplative science research. This fundamental assumption might be at the core of the challenges contemplative science is currently facing. For example, if mindfulness (or any other contemplative practice) as a practice, capacity, process, or outcome is the main subject of contemplative science, the definitional difficulties associated with the term ‘mindfulness’ are of principal importance. However, if the main subject of contemplative science is defined in terms of an overarching (practice non-specific) capacity, process or state of the mind, the terminological problems arising from disparate definitions of contemplative practices are of secondary importance – difficulties of terminology associated with defining practices can be greatly reduced by clear instructional descriptions of particular practices and processes or outcomes they aim to produce. Similarly, if contemplative science is defined in terms of mental or cognitive constructs non-specific to particular contemplative practices (to avoid equating the discipline and its subject with contemplative practices), this would focus investigations on underlying similarities and differences (in terms of mechanisms) across practices, thus contribute to advancement of an integrative contemplative science theory. In addition, defining the subject in terms of mental or cognitive constructs could create theoretical bridges enabling focused considerations about the unique potential of contemplative science in contributing more broadly to research in cognitive science and psychology. Finally, such definition of contemplative science could also help clarify the methodological focus of the discipline – if the formulation of the discipline’s subject clearly highlights the importance of studying the experiential dimension of mental functioning and context of contemplative practice, this will necessitate the integration of first- and second-person approaches and considerations about contemplative context into its core methodology. In line with this approach, this paper proposes one possible definition of contemplative science and its subject.

## Defining Contemplative Science

### The Metacognitive Self-Regulatory Capacity of the Mind

#### Self-Regulation and Contemplative Practice

**FIGURE 1:**
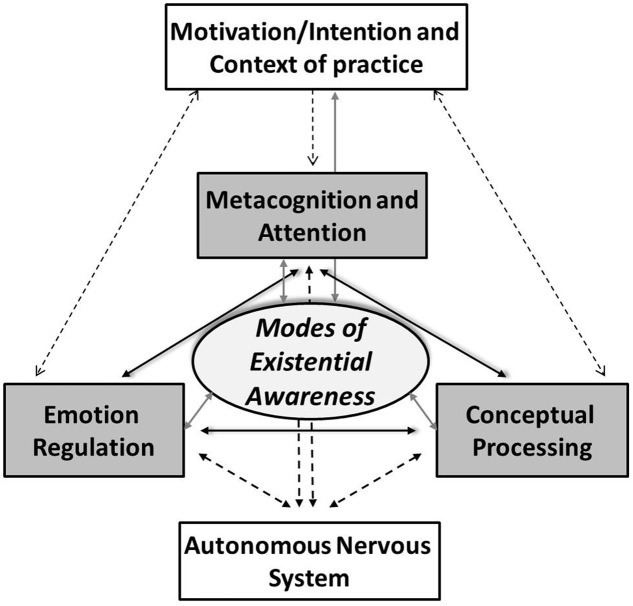
**Core systems and processes modified by contemplative training.** The metacognitive self-regulatory capacity of the mind (MSCM) consists of interacting systems and processes of metacognition and attention, emotion regulation, and conceptual processing. Cumulative changes in the MSCM enable shifts in modes of existential awareness (MEA). Both changes in the MSCM and shifts in MEA are modulated by motivational/intentional and contextual factors of contemplative practice. Motivational/intentional factors can also be impacted by the shifts in MEA, and to some extent modulated by changes in the MSCM. Changes in the MSCM and associated shifts in MEA further modify the sympathetic/parasympathetic balance of the autonomic nervous system (ANS) which mediates their impact on physical well-being. Mostly via the neuroendocrine mechanisms of the hypothalamic–pituitary–adrenal (HPA) axis, the ANS changes also impact Metacognition and Attention, Emotion Regulation, and Conceptual Processing.

Enhancement of self-regulation (SR) as the ability to notice and effectively manage thoughts, emotional responses, and behavior has been highlighted as the main neurocognitive mechanism of mindfulness – the most studied meditation technique (Hölzel et al., 2011; Tang et al., 2015). Setting aside the widely debated differences in definitions of mindfulness (‘sati’ in Pali), the core SR processes of orienting, shifting, and sustaining attention combined with metacognitive awareness (‘sampajanna’ in Pali, Thera, 1998; Wallace, 1999a) are emphasized in most definitions of mindfulness. They are also considered essential pre-requisites for any meditation practice in the Buddhist context. This makes the construct of SR broadly applicable to all contemplative research. However, SR is also a construct used within and outside of contemplative research, which highlights the need to specify how meditation practices could distinctively engage SR (**Figure 1**).

Self-regulation is in psychological research typically defined in terms of adaptive goal-directed attention regulation (including directing, shifting, and sustaining of attention; Hofmann et al., 2012) and emotion regulation (enabling modulation of responses to emotional stimuli; Gross and Thompson, 2007) – processes essential to effective modulation of behavior and in social interactions.^2^ Such definition of SR seems equally relevant to contemplative science research, but contemplative training particularly emphasizes a specific aspect of SR rarely considered in psychological research – the introspective metacognition (awareness and knowledge of bodily sensations, mental phenomena, and behavior). This is where many meditation techniques seem to engage SR distinctively from other methods of SR training since noticing immediate thoughts, feelings, perceptions, affect, and behavior, as well as noticing overarching patterns of these is their hallmark.

#### Emotion Regulation in Contemplative Context

Another aspect of SR particularly targeted by contemplative practices aims to develop specific emotional qualities and emotion regulation strategies. These emotion regulation processes closely build on the skills of attention control, sustained attention, and metacognition and also impact these (see links between Metacognition/Attention and Emotion Regulation in **Figure 1**). The qualities of acceptance and non-reactivity are most often highlighted in meditation research (e.g., Bishop et al., 2004; Baer et al., 2008) together with positive emotions of loving kindness and compassion (e.g., Hofmann et al., 2011). Some traditions equally emphasize sympathetic joy and equanimity (Wallace, 1999b). Contemplative training in cultivation of these qualities and emotions typically encourages development of unique emotion regulation strategies involving affective labeling, visualization, reflective contemplations, transformation of negative emotions into their positive emotion ‘antidotes,’ etc. There are many important differences across contemplative traditions in terms of their emphasis on particular qualities, emotions, and strategies. There are also differences in terms of practices leading to their cultivation (e.g., secular mindfulness-based approaches train in loving kindness and compassion mostly implicitly; Tibetan Buddhist schools use a variety of explicit conceptual and visualization-based practices training different degrees of loving kindness, compassion, rejoicing, and equanimity; and distinct practices, some of them visualization-based, are applied in Christian contemplative training of the Ignatian tradition). These variations deserve much more attention in research than they have received so far, but the overarching emphasis on cultivation of certain positive affective states and traits is another feature of SR unique to this construct within the contemplative science context.

#### Conceptual Processing, Self-Regulation, and Contemplative Practice

A further aspect of SR, which is typically not considered in psychological models of SR, and rarely considered in meditation research, is the contribution of language processes, and more broadly conceptual processing, to SR. For instance, Farb et al. (2010) found that novices to meditation showed higher activation in left prefrontal and Wernicke regions of the brain (possibly due to rumination) associated with higher depression scores in comparison to participants who completed a mindfulness-based stress reduction (MBSR) course. In a cross-sectional study, Pagnoni et al. (2008) reported less activation in areas involved in conceptual processing in Zen meditators in comparison to non-meditators in a lexical decision task. And in a dispositional study Dorjee et al. (2015) found a positive association between trait mindfulness and the N400 effect to negative targets of affective word pairs suggesting less frequent access of negative word meanings in more mindful individuals.

Such changes in language processing might be viewed as consequential to enhancements in attention and emotion related SR. However, they could equally reflect distinct engagement with conceptual processing targeted by contemplative practices, for example when meditation encourages non-elaborative awareness of thoughts (with thoughts most often overtly and covertly expressed in language), which could release more cognitive resources for SR. In addition, some meditation techniques involve focused contemplations on topics such as impermanence (involving overt or silent speech), and other meditations teach neutral language for labeling of experience, use mantra recitations, or encourage complex non-verbal visualizations. These targeted modulations of language/conceptual processing most likely uniquely contribute to the SR changes resulting from meditation. Therefore, conceptual processing needs to be singled out as one of the mechanisms of SR modified by meditation and can both be directly impacted by and directly impact on metacognition, attention, and emotion regulation processes (see links between Conceptual Processing, Metacognition, and Attention and Emotion Regulation in **Figure 1**).

#### Motivational/Intentional and Contextual Factors

Self-regulation in the context of meditation research is also intrinsically linked to motivational/intentional^3^ and contextual factors (Shapiro et al., 2006; Dorjee, 2010; Vago and Silbersweig, 2012). For instance, some practitioners may engage in contemplative practice to learn how to cope with stress or better regulate chronic pain, whilst for others engagement in contemplative practice may be a way to find existential meaning and purpose. Importantly, the motivational/intentional factors are in the contemplative context closely linked to the broader philosophical context of a practice and particularly to ethics – cultivation of states and qualities considered virtuous within a specific contemplative framework. Dahlsgaard et al. (2005) suggested possible convergence on a few core virtues across contemplative traditions, but there are inevitably also important differences. Some contemplative traditions include clear specifications of faculties, qualities, and states as virtuous, non-virtuous, and neutral (Thera, 1998). Contemplative training accordingly targets development of virtuous qualities and states, and reduction of non-virtuous ones (Dreyfus, 2002), as determined by broader motivational/intentional factors.

The specification of motivational/intentional factors is often closely linked with broader context of contemplative practice, most notably practicing within a certain tradition with its specific philosophical views shaping the perceptions and goals of the practice. Another salient aspect relates to practicing within a structured ordained contemplative context which typically focuses on explicit motivational/intentional factors (whether monastic or non-monastic), whereas outside of structured context of traditional contemplative training the motivational/intentional factors are typically less specified and emphasized. While some of these motivational/intentional and contextual factors have been theoretically highlighted in meditation research (Shapiro et al., 2006; Dorjee, 2010, 2013), they are rarely considered in experimental studies evaluating effectiveness and mechanisms of meditation. Our understanding of the progression of motivational/intentional factors with long-term contemplative practice is also very limited (e.g., Shapiro, 1992). In some contemplative traditions such as in Mahayana Buddhism, a progression from self-focused to self-transcending motivation/intentions is explicitly encouraged (Dorjee, 2013). Overall, the motivational/intentional and contextual factors likely significantly modulate practitioners’ engagement with contemplative practices and resulting changes in SR (see links with Metacognition and Attention, Emotion Regulation and Conceptual Processing in **Figure 1**). Modulations in SR, particularly changes in conceptual schemas relevant to self-construal and values, can also impact on motivational/intentional factors.

#### Postulating the Metacognitive Self-Regulatory Capacity of the Mind

In sum, contemplative practices seem to distinctively improve attention and introspective metacognition^4^ of mental contents/processes and behavior. They also cultivate particular affective states, traits, and emotion regulation strategies, and uniquely modify conceptual processing. These cognitive, affective, and conceptual states, traits and processes can be considered features of an overarching natural capacity of the mind, specifically termed here ‘the metacognitive self-regulatory capacity (MSRC) of the mind,’ which contemplative training aims to enhance. This capacity enables reflective meta-awareness of mental phenomena and behavior and their adaptive^5^ (well-being conducive) modulation. The processes of the MSRC are modified by motivational/intentional and contextual factors of contemplative practices (**Figure 1**).

While the MSRC is considered here as an umbrella term, the particular pattern of changes in the MSRC resulting from specific contemplative training will likely differ across contemplative practices and traditions. All meditation practices enhance to some degree metacognitive awareness and attention control since these are foundational to any meditation practice. But contemplations on a certain topic (e.g., impermanence) may also distinctively engage language systems with implied impact on motivational/intentional factors and emotion regulation. In comparison, visualization-based practices may enhance visuo-spatial conceptual processing (e.g., Kozhevnikov et al., 2009) and reduce activation in language areas with indirect impact on emotion regulation in a broader spiritual development framework. Mindfulness-based approaches may modify involvement of language areas through their emphasis on non-elaborative processing and sensory focus (e.g., Farb et al., 2010), and more directly target development of specific adaptive emotion regulation strategies in the secular context, etc.

#### Modulations of the Autonomic Nervous System

Enhancements in the MSRC resulting from contemplative practice can also have tangible effects on physiological processes associated with well-being and health (see links between Metacognition and Attention, Emotion Regulation, Conceptual Processing, and autonomic nervous system (ANS) in **Figure 1**). These have been so far mostly studied in terms of modulations in the sympathetic/parasympathetic balance of the ANS linked to hypothalamic–pituitary–adrenal (HPA) axis activation during the stress response. The initial evidence suggests that two main indexes of both acute and chronic stress might be modulated by contemplative practice – these are cortisol levels as a marker of endocrine stress response and heart-rate variability (HRV) indexes of sympathetic/parasympathetic balance. For example, amount of meditation practice has been negatively related to the levels of morning cortisol suggesting less chronic stress (Brand et al., 2012). However, findings on the effects of MBAs on cortisol levels are mixed (O’Leary et al., 2015). Initial findings on changes in HRV indexes reported increases in high frequency HRV, a biomarker of parasympathetic activity, after 10 days of Vipassana training (Krygier et al., 2013).

Yet, very few studies examined actual links between modulations in ANS activity and neurocognitive processes of the MSRC with contemplative practice – a study by Tang et al. (2009) was one of rare exceptions. They found an association between an EEG index of ACC activity and high frequency HRV in a group trained in integrative mind-body training (which includes mindfulness practices). No studies have so far explored how sympathetic/parasympathetic balance as such could impact on engagement with contemplative practice and associated changes in the MSRC (see reciprocal links with ANS in **Figure 1**). Similarly, no studies investigated links between ANS activation and intentional/motivational and contextual factors of contemplative practices.

Investigation of similarities as well as differential modulations of the MSRC (its processes and outcomes) by contemplative practices together with their motivational/intentional and contextual factors and their implications for modulations of the ANS balance can be considered one of the two essential goals of contemplative science. The second (not less important) goal of contemplative science is to investigate phenomenological shifts in the awareness of self and reality resulting from contemplative practices. We will now examine the current empirical evidence and theoretical foundations of these meditation-specific changes in awareness.

### Modes of Existential Awareness

#### Changes in Self-Construal with Meditation: Current Approaches and Their Limitations

Modifications in the awareness and construal of self and reality are often highlighted as the essential aims of contemplative practices across traditions (Dahl et al., 2015). Yet, Western scientific understanding of associated processes, states, and traits is very limited. For example, Farb et al. (2007) investigated changes in self-referential awareness by comparing narrative construal of self with present-moment sensory experience focus in participants who completed an MBSR course and novices to meditation (Farb et al., 2007). The findings revealed increased activation in the right insular cortex and somatosensory cortex for the mindfulness group in the present-moment condition. This suggests differential activation between the narrative and present-moment modes which seems enhanced further by mindfulness training. It is, however, not clear to what extent these findings mostly reflect differences in attention focus (bodily sensations vs. narration) during the two instructions rather than differences in the actual construal of self.

Perhaps closer to investigating specific shifts in self-construal with meditation, a recent study examined neural correlates associated with non-reactivity to self-praise and self-criticism in long-term mindfulness meditators and meditation novices (Lutz et al., 2016). The results showed increased activation in dorsomedial prefrontal cortex (possibly reflecting greater regulation of emotions) and decreased connectivity between this area and other regions in the default mode network in meditators compared to novices. The later finding was interpreted as an indicator of less self-focus in meditators, manifesting as less reactivity. The authors, however, acknowledge the difficulty in distinguishing between emotion-specific and self-referential effects in their results and argue that these might be closely interconnected, so are hard to tease apart.

Other meditation studies evaluated more spontaneous self-related processing by measuring the default mode of brain function (Raichle et al., 2001; Raichle and Snyder, 2007) which is typically assessed using functional magnetic resonance imaging while participants are simply resting or fixating their gaze on a cross on the screen. This encourages engagement in spontaneous ‘default’ mode processing which for most people seems to involve mind-wandering (random off task activity). In a study with experienced meditators and meditation novices, Brewer et al. (2011) found that during resting as well as during meditation, meditators showed stronger connectivity between the posterior cingulate, dorsal anterior cingulate, and dorsolateral prefrontal cortices. This suggests better self-monitoring and cognitive control in meditators. In addition, findings of deactivation in the core areas of the default mode network (medial prefrontal and posterior cingulate cortices) during resting and during three different meditation practices (concentration, loving kindness, choiceless awareness) were interpreted as less mind-wandering in meditators. Similarly, Taylor et al. (2013) found that meditators had decreased connectivity between medial prefrontal cortex (associated with mind-wandering and self-referential processing) and other regions. While these results are certainly novel and interesting, again, it is not clear what aspects of the brain activation differences are distinctively relevant to self-construal and which reflect more overarching attention processes. Particularly the result of no differential modulations across three meditation types and the resting state in the Brewer et al. (2011) study indicates that the brain activation pattern most likely reflected similar regulation of attention processes, rather than self-referential processing. This is because the practice of choiceless awareness closely targeting self-related processes would be expected to more selectively reduce self-construal related activity than focused attention (Lutz et al., 2008).

So despite the increasing numbers of studies investigating modulations in self-referential processes with meditation, implications of these findings for our understanding of changes in the self-construal resulting from meditation seem limited. The observation of overlap between brain activations associated with emotion and self-reference in research on meditation (Lutz et al., 2016), just like the difficulty in dissociating attention (Farb et al., 2007) or mind-wandering (Taylor et al., 2011; Brewer et al., 2013) and self-referential processes in other meditation studies, raise a key question for this type of research in contemplative science: Is the construal of self a mere accumulation of other, mostly attention, emotion, and memory related processes? This is the approach commonly applied in cognitive and neuroscientific research on self-reference (Northoff et al., 2006), and most current meditation studies seem to assume that it is equally appropriate for investigating changes in construal of self with contemplative practices. But some contemplative traditions provide descriptions of radical deconstructing of the usual self-referential processing with contemplative practices. Can such changes be meaningfully captured using the traditional cognitive and neuroscientific methods?

A study by Josipovic (2014) was the first aiming to investigate the more advanced shifts in self-focus by examining neural correlates of awareness states characterized by greatly diminished differentiation between subject and object of experience (non-duality) in experienced meditators. The findings indicated clear differences between brain activation patterns in focused meditation and non-dual awareness meditation, thus dissociating the two. However, once more it is not clear to what extent these differences were the result of implementing distinct types of meditation with different objects of attention focus. The main result highlighted an increase in functional connectivity between the central precuneus and dlPFC which was interpreted as a possible index of gradients in non-dual awareness. This hypothesis might be so far the closest to specific postulation of neural changes associated with modulations in the self-construal with meditation.

Perhaps the lack of research evidence directly pertaining to the changes in self-construal with meditation is due to one key aspect of these states being omitted in the majority of previous investigations of self-referencing in meditators. This component relates to the experiential felt ‘what it is like’ aspect of changes in the self-construal. If we capture the phenomenological shifts in the self-construal, we might be able to investigate the core distinctive features of self-construal states rather than attention, emotion, memory, or other processes which support the self-referencing states, but are not specific or definitional to them. Such phenomenological reports would enable the assessment of links with modulations in behavioral or neural markers, while contributions of emotion, attention, or other cognitive processing are controlled for. A recent study by Dor-Ziderman et al. (2016) was the first step toward this approach; it captured the phenomenological aspects of self-construal by inviting meditators to describe their sense of self-boundaries and being separate from external world. Associations between three distinct self-construal states and neural correlates were then investigated using magnetoencephalogram, with findings highlighting changes in the right lateralized beta oscillations in the temporo-parietal junction and in the medial parietal cortex. However, given the lack of an overarching theory of phenomenological states associated with self-construal, it is currently difficult to position these findings within a range of such states resulting from meditation training.

Hence, an essential pre-requisite for future testing of any hypotheses about changes in the self-construal with meditation seems to be a systematic capture of first-person phenomenological reports on gradients of self-construal grounded in a comprehensive theory of such states. This approach will necessitate further development of suitable first-person and second-person research methods which would be particularly suited for research in contemplative science (see section “Definitions of Mindfulness and Other Contemplative Practices” for further discussion). It will also require development of a comprehensive theory which would describe differences in self-referential processing expected with increasing proficiency in specific types of contemplative practices, and how these shifts could relate to self-regulatory processing and well-being. Such theory would provide grounding and guidance for further systematic and focused empirical investigations of changes in the construal of self with meditation.

#### Postulating Modes of Existential Awareness

In the proposed framework of contemplative science it is suggested that cumulative changes in the attentional, affective, and conceptual aspects of metacognitive self-regulatory processes modulated by motivational/intentional and contextual factors of meditation practice enable more overarching, phenomenologically distinct, state shifts in the awareness of self and reality (**Figure 1**). These shifts are termed here ‘modes of existential awareness’ (MEA) to encompass both the experiential modulations in the construal of self and in experiential understanding of the construed nature of reality. They phenomenologically reflect personal existential schemas of meaning and purpose in life, that’s why they are best described as ‘existential.’ Meditation training is associated with a progression of shifts in MEA. For example, in the Buddhist context of the Dzogchen tradition, the initial MEA are associated with the ‘ordinary mind’ characterized by a construal of self and reality which is heavily determined by our cognitions, habits, personal history, culture, and society. The same tradition describes that at the most advanced stages of meditation training the MEA involve insight into the nature of mind and reality characterized by experiential recognition/knowing and abiding in experiential ground from which self and reality are construed (‘rigpa’ – pristine awareness, Gyaltrul and Wallace, 1998; Padmasambhava, 2007).

**FIGURE 2:**
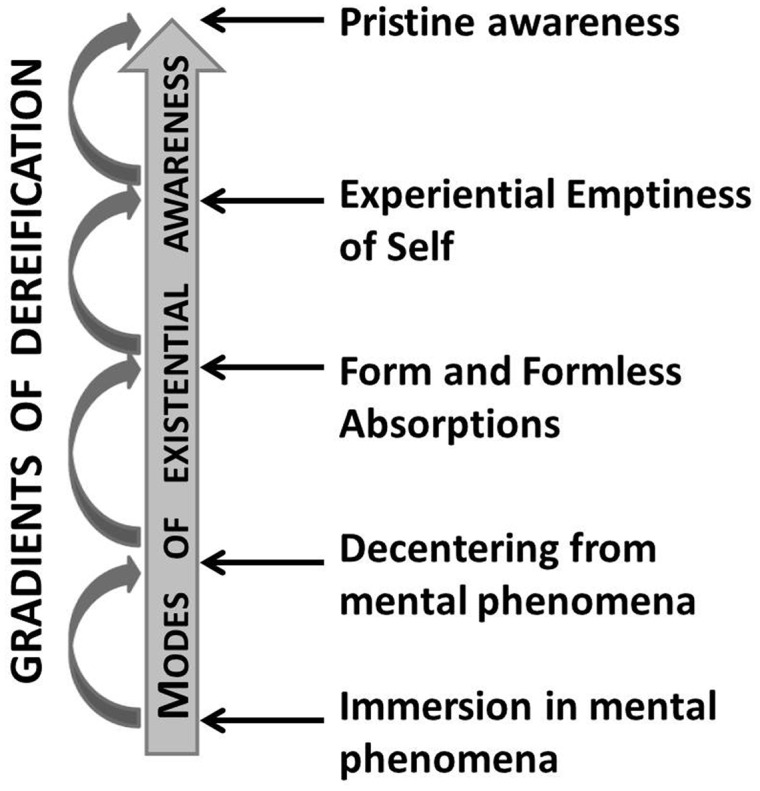
**The figure outlines the progression in MAE as states associated with increasing gradients of dereification (processes).** Further MAE can be described between the MAE states highlighted in the figure (together with associated dereification gradients). Some types of contemplative training may bypass certain MAE (e.g., Tibetan Buddhist traditions typically do not engage in the training of the form and formless absorptions).

There are many additional MEA between the two extreme end points (**Figure 2**). These could involve a progression of states characterized by increasing development of self-reflective awareness and self-inquiry of the fleeting nature of mental phenomena, then first intellectual understanding of the construed notions of self, followed by experiential realization of the emptiness of self and finally non-dual state of pristine awareness (Dorjee, 2013). Looking more closely at the underlying processes, the accumulation of the initial experiences of impermanence can lead to loosening up of the usual self-construal which is at first still rooted in conceptual distinctions about what self is and isn’t. This is enabled by increasing levels of stability in MSCM, which in some meditation traditions culminates in the experience of form and formless absorptions (Sanskrit: Dhyāna; Pali: Jhāna). Such states, however, are not considered as stages of actual liberation because of their focus on concentration rather than insight into the nature of self and reality (e.g., Gampopa, 1998).

The emptiness of ‘self’ is further realized through purely experiential comprehension which releases the conceptual constructs of self and non-self. The most advanced mode of existential awareness (in Dzogchen termed as rigpa – pristine awareness; Dalai Lama, 2000) experientially transcends the notions of self through the dissolution of the duality between the observer and the observed and encompasses the experiential realization of the nature of mind and reality. It is a non-conceptual state with a clear sense of knowing (which can be considered paradoxical from the ‘ordinary mind’ perspective where all knowledge is conceptual) and pervasive non-referential experience of compassion. One of the key future tasks of contemplative science is to provide a clear theory of the MEA progression which could then be empirically tested. Such research needs to emphasize the intrinsically phenomenological nature of MEA and take into account the limitations of research methodologies in capturing the non-conceptual nature of more advanced MEA.

The only MEA which has so far been investigated in meditation research is decentering which could be considered one of the initial stages in the MEA progression. Decentering (Fresco et al., 2007) has been described as the awareness of mental events experienced as observing thoughts and feelings as fleeting mental phenomena rather than unchangeable facts. Decentering plays pivotal role in therapeutic effects of mindfulness-based approaches (Bieling et al., 2012; Hoge et al., 2015), and is particularly emphasized in mindfulness-based cognitive therapy (MBCT; Teasdale, 1999). Specifically, in research on recurrent depression the term ‘metacognitive insight’ has been used to label this therapeutically salient shift in individual perspective on elements of own cognition (Teasdale et al., 1995, Teasdale et al., 2002). In a similar way, Shapiro et al. (2006) and Carmody et al. (2009), use the term ‘reperceiving’ to denote the modification in perspective on mental events and suggest that reperceiving is the core primary mechanism underlying the beneficial effects of mindfulness with other clinically significant mechanisms such as exposure or changes in SR and cognitive flexibility being derivative. Recent psychophysiological research partially supports this hypothesis – Eddy et al. (2015) found that decentering as a state (but not mindfulness) was associated with less demand on cognitive resources in processing of emotional images in healthy adults. It is also possible that even within the construct of decentering we could differentiate further distinct MEA, this option has not been investigated so far. Similarly, it is not clear whether secular mindfulness-based approaches could enable a progression beyond initial stages of MEA.

#### Conceptual Processing and Modes of Existential Awareness

Enhancements in the MSRC of the mind can be considered pre-requisites for all shifts in MEA since these shifts require increasing levels of attentional stability, cultivation of certain affective qualities and emotions (such as compassion and sympathetic joy), clarification of personal motivation/intention for engaging in contemplative practice and volitional monitoring and regulation of elaborative conceptual processing.^6^ The contribution of conceptual processing to shifts in MEA is likely pivotal in this process and is not limited to improved volitional control of rumination (as a support of non-elaborative awareness, discussed in section “The Metacognitive Self-Regulatory Capacity of the Mind”). Concepts are the fundamental building blocks of cognition and of particular importance here is the ‘intentionality of concepts’ as their property of representing entities whilst being detached from them. In the context of contemplative research John Dunne (2012) proposed the term ‘dereification’ to describe the explicit experiential awareness of the distinction between the actual objects and conceptual representations of the objects. Increasing gradients of dereification, starting with enabling initial decentering from mental states up to the most advanced dereification of ways we conceptualize reality, can be hypothesized as the main mediators (or underlying processes) of shifts in MEA (**Figure 2**).

The conceptual changes mediating shifts in MEA can to some extent be further elucidated by a theory underlying the postulation of metacognitive insight in MBCT which builds on the Interactive Cognitive Subsystems Framework (ICS) (e.g., Barnard and Teasdale, 1991). The ICS distinguishes between specific propositional meanings expressed in language and more holistic, language non-specific, implicational meanings described as generic schematic experiential patterns, with both types of meanings continuously interacting.^7^ Importantly, Teasdale (1999) linked this distinction to metacognitive knowledge supported by propositional meanings and metacognitive insight which can only arise at the level of implicational meaning representations. In the context of MBCT, through metacognitive insight a practitioner develops a new implicational schema for mapping perceptions and thoughts onto reality which can reduce depressive relapse through decentering from negative propositional meanings and implicational schemas. In other words, metacognitive insight enables initial level of experiential understanding of the ‘intentionality of concepts.’ This supports the development of a mode of existential awareness associated with initial stages of dereification. Beyond the context of MBCT and with further contemplative training, particularly in the deconstructive family of meditation practices (Dahl et al., 2015), a practitioner can experience shifts toward more advanced MEA encompassing further remapping of implicational meanings associated with deeper experiential understanding of dereification.

#### Are Modes of Existential Awareness States or Traits?

The links between gradients of dereification (considered here as processes) and MEA (which have been here so far described as states), raises the question about stability of these shifts. Are they only transient insights or could they become more stable states and traits? In the traditional contemplative context, they can be both depending on depth and stability of one’s contemplative practice (Dorjee, 2013). Most novices to meditation practice may experience initial insights of decentering within few weeks or months of their practice, but will not be able to stabilize the state. Further deepening and stabilization of increasingly advanced shifts in MEA will come with ongoing practice under proper guidance. There can also be instances of sudden immediate insight into most advanced MEA, such as those experienced in ‘cutting through’ practices of Dzogchen, but for most practitioners these too will need to be stabilized and sustained with further practice up to the point where they can be considered a stable trait. Such stabilization can happen at any mode (also considered here as a stage) of existential awareness associated with any degree of dereification (including lack of it which would be associated with stagnation in progress to more advanced MEA).

In general, the progression in MEA with further practice is in contemplative theory associated with higher levels of well-being. However, progression from one MEA onto another, more advanced one, can also sometimes be accompanied by temporary psychological states which could be in the Western psychological or psychiatric context considered adverse or pathological. Such states are clearly outlined in the contemplative literature as temporary signs of shifts in meditative experience together with instructions on how to work with such experiences (e.g., Wallace, 2011). This highlights the essential importance of guidance by experienced qualified meditation teachers when engaging in practices targeting the progression of MEA in one’s contemplative training.

Overall, investigation of the shifts in MEA with meditation training has perhaps been the most elusive part of research in contemplative science so far, yet it seems the most central and unique to the discipline. The proposed framework aims to stimulate further investigation in this underexplored area of contemplative science research through postulating initial theoretical distinctions in MEA and their mediation by dereification of intentionality of concepts. The modulation of MEA by motivational/intentional and contextual factors will be of particular interest in future studies, especially in investigations of similarities and differences in MEA across contemplative approaches. Given the central standing of shifts in MEA in traditional contemplative training, such investigations could provide impactful new insights advancing contemplative science theory with further implications for applied research.

### The Proposed Definition of Contemplative Science

The discussion in preceding sections highlighted the necessity of defining contemplative science in terms of overarching cognitive and mental capacities, processes and states – the MSRC of the mind and MEA were considered as the possible key definitional constructs. These considerations imply that contemplative science can be defined as an integrative interdisciplinary study of the MSRC of the mind (which contemplative training aims to enhance) and associated MEA (states and traits of awareness resulting from contemplative training characterized by increasing gradients of dereification), with both modulated by motivational/intentional and contextual factors of contemplative practice. Changes in conceptual processing, particularly in implicational meanings, are likely mediators between changes in the MSRC and shifts in MEA. First- and second-person methods will be essential to mapping the shifts in MEAs and conceptual processing, hence are central to the methodology of contemplative science. We will now explore the methodological implications of the proposed definition in more detail.

### Research Methodology of Contemplative Science

Contemplative science is inherently an interdisciplinary field of study due to the multifaceted nature of its subject which includes both complex mental processes and states and their contemplative context involving historical, cultural, and societal factors. So far, meditation research has been mostly building on standard research methods in psychology and cognitive neuroscience. This approach seems appropriate for the core components of the MSRC of the mind – attention and emotion regulation, and conceptual processes. It is also suitable for investigations of the impact changes in MSCM can have on the balance of the ANS. From amongst the components of MSRC, previous research on meditation mostly studied attention processes and emotion regulation (e.g., Hölzel et al., 2011; Tang et al., 2015). Studies on changes in introspective metacognition (e.g., Teasdale et al., 2002; Jankowski and Holas, 2014) and conceptual processing with meditation (e.g., Pagnoni et al., 2008; Dorjee et al., 2015) are, however, limited.

For research in contemplative science to move toward a more comprehensive contemplative science theory, the modulations in processes of MSRC need to be studied systematically and simultaneously whenever possible. In most studies this is a realistic option since multiple experimental tasks and self-report measures are often used in a single experimental session with same participants; yet outcomes from different tasks are then typically divided into separate research reports. There is a need for a more systematic selection of tasks driven by contemplative science theory so that these assessments would systematically target the core elements of the MSRC. In addition, separate reports of findings from the same participants should make the links across such studies clear to enable integration of findings across assessments. Comparison and consideration of broader implications of findings across experiments with the same participants could be instrumental in building a more comprehensive and integrated picture of modulations in MSRC with contemplative practices.

The proposed framework of contemplative science also necessitates an expansion of the methodological and theoretical scope of contemplative research to include motivational/intentional and contextual modulators of processes and outcomes. Currently, studies on motivational/intentional (e.g., Shapiro, 1992) and contextual factors (Christopher et al., 2009) are very scarce. Further research will require development of assessment tools and experimental tasks which will particularly target investigation of these factors in the contemplative science context. This is because the available psychological and neuroscientific tools are limiting in assessment of the intentional/motivational and contextual aspects of contemplative practices since the dimensions of these factors (e.g., intention for liberation from suffering, spiritual transcendence, broader philosophical context, secular or religious context, etc.) have not been sufficiently investigated in the more established disciplines. Some of the new methods will need to build closely on traditional contemplative theories and anthropological methods applied to examine cultural, philosophical, and societal dimensions of contemplative practices. As a result, future studies in contemplative science will, hopefully, meaningfully combine psychological and neuroscientific methods with philosophical, anthropological, and traditional contemplative theories and methods as a rule, rather than as an exception. At the current stage, it would be advantageous if each study included, at minimum, a clear description of instructions used in a contemplative practice studied, outline of processes (psychological or cognitive) the practice aims to modify, and outcomes the practice aims to produce within a particular contemplative context (see section “Definitions of Mindfulness and Other Contemplative Practices” for more details).

It has been repeatedly emphasized that first-person methodologies need to play an essential role in research on meditation (Varela et al., 1991; Lutz, 2002; Nielsen and Kaszniak, 2007). Neurophenomenological approach suggesting integration of immediate first-person introspective input and third-person neurocognitive data in experimental tasks (e.g., Lutz, 2002) has been proposed as a possible methodological solution. Yet, this recommendation has been rarely followed in empirical research. One of the reasons might be the complexity of neurophenomenology and lack of examples which would encourage others to apply the same approach. It is also possible that in the absence of a clear definition of contemplative science which would stipulate the central standing of first-person methodologies in the discipline, the research easily bypassed this ‘methodological complication.’ The proposed framework of contemplative science further highlights the primary methodological importance of first-person methodologies in contemplative research, particularly due to its emphasis on the intrinsically phenomenological nature of shifts in MEA. It also raises the question whether these phenomenological shifts can be reliably investigated with available introspective methods, including neurophenomenology.

Standard qualitative methods such as interviews or diaries analyzed using traditional qualitative methods (e.g., thematic analysis, interpretative phenomenological analysis, discourse analysis, etc.) may not be most suitable to investigate shifts in MEA. This is due to the intrinsically private and unique nature of the experiential shifts associated with MEA which would be subject to morphing and reshaping by an ‘outsider’ interpreter without access to the primary phenomenological data. Temporal delay in descriptions and interpretation of such states can be another source of misrepresentation (e.g., Schooler, 2002). The neurophenomenology approach is a step forward in this regard because it highlights the importance of temporal accuracy of reports and involvement of meditators as collaborators in contemplative science research. However, the neurophenomenological approach is specifically suited for lab investigations with experimental tasks, and there is also a need for methods which would enable capturing MEA as traits manifesting in real life, outside of experimental environment.

There is a need for new methods which would invite phenomenological report and self-interpretation by the participant herself, inside and outside of experimental environment, and without a temporal delay. An example of one possible methodological approach satisfying such requirements is the SenseMaker^8^, but it is yet to be seen to what extent this approach is applicable in contemplative science. Second-person methods can also play a pivotal role in MEA research since meditation experts, based on their personal extensive experience with MEA, are best positioned to provide an informed ‘outsider’ interpretative input. And even approaches relying on self-interpretation of first-person data and on second-person expert data need to acknowledge the inevitably indirect nature of findings resulting from any research method which attempts to describe non-conceptual MEA in words or other conceptual format. Overall, the unique standing of contemplative science amongst other disciplines could be strengthened if its research followed a stronger theory-driven interdisciplinary approach which effectively integrates evaluations using psychological and neuroscientific methods with assessments of motivational/intentional and contextual factors, and first-/second-person methods whilst acknowledging their limitations.

## Implications of the Proposed Definition

### Definitions of Mindfulness and Other Contemplative Practices

In the secular context of mindfulness-based approaches (MBAs – including MBSR and MBCT), mindfulness is most commonly defined as ‘the awareness that emerges through paying attention on purpose, in the present-moment, and non-judgmentally to the unfolding of experience moment by moment’ (Kabat-Zinn, 2003, p. 145). Further considerations building on this definition operationalized mindfulness in terms of attention and attitude (emotion qualities of openness, curiosity, non-judgment, etc.) as its two main components (Bishop et al., 2004). Shapiro et al. (2006) added to these two components a third one – intention of mindfulness practice. Finally, in one of the most recent definitions, mindfulness was specified as developing self-awareness, SR, and self-transcendence (S-ART, Vago and Silbersweig, 2012). These definitions will now be considered further in the context of the outlined contemplative science framework.

From the perspective of the proposed framework, the definition by Kabat-Zinn seems to equate secular mindfulness with a particular type of awareness, but the definition does not specify further characteristics of this state. In the literature on MBAs, the reference to awareness has been used in varied connotations, ranging from simple noticing of the contents and processes of mind to states of decentering from them (e.g., metacognitive insight in Teasdale et al., 2002) and self-transcendence (Vago and Silbersweig, 2012). Across these different meanings, changes in awareness seem to be consistently considered as outcomes of mindfulness training. Such changes could be examined and further specified in terms of the progression in MEA with contemplative training. It seems that training in MBAs may specifically result in the initial modulations of MEA associated with decentering; it is not clear though whether MBAs can enable progression onto further stages of MEA.

It is also not clear whether the changes in awareness are the sole result of the mindfulness training as part of MBAs, or whether they are cumulative outcomes of mindfulness practices and other aspects of training in MBAs – such as implicit development of self-compassion, group therapy effects, acceptance elements of practices, etc. The literature has so far been mostly equating the effects of mindfulness with the effects of the MBAs. It would certainly be expected that the effects of mindfulness developed in MBAs and effects of MBAs as multifaceted interventions, would be overlapping, but future research may reveal that their underlying mechanisms and outcomes are somewhat different. It is also up to future studies to investigate whether, in the secular context, the changes in decentering are specific to MBAs, or whether they can be cultivated through other secular contemplative practice programs, particularly those targeting development of compassion and self-compassion.

While changes in awareness with training in MBAs can be considered their outcomes, the processes of mindfulness are typically described in terms of attention and emotion regulation. This is exemplified by the reference to the present-moment attention with a non-judgmental attitude in the Kabat-Zinn definition; the definitions by Bishop et al. (2004) and Shapiro et al. (2006) specifically single out attention and attitude as the core components of mindfulness. According to the proposed conceptualization of contemplative science, metacognition/attention and emotion are the essential aspects of the MSRC of the mind. However, this framework postulates that all contemplative practices modify the MSRC, yet they differ in terms of specific patterns of resulting changes in the components of the MSRC (see section “The Metacognitive Self-Regulatory Capacity of the Mind” for examples). The distinctive features of mindfulness developed in MBAs likely relate to developing metacognitive awareness (noticing) of mental contents and processes, enhancement of attentional stability and cultivation of the attitude of non-reactivity.

Alongside attention and emotion processes, secular mindfulness definitions also highlight the importance of intention to the mindfulness practice. Kabat-Zinn (2003) refers to this in his definition by the phrase ‘on purpose’ and Shapiro et al. (2006) explicitly named intention as one of the three components of mindfulness in MBAs. Vago and Silbersweig (2012) also included intention as one of the components of mindfulness and discussed its relation to motivation. However, the postulated framework of contemplative science considers motivational/intentional factors as non-specific to a particular practice, impacting on processes and outcomes of any contemplative practice. This implies that inclusion of intention-related components (just like awareness-related outcomes) in definitions of mindfulness needs to be practice-specific, indicating how the intention in mindfulness practice within MBAs would be similar or different from the motivational/intentional factors in other contemplative practices.

Based on the postulated definition of contemplative science, contemplative practices need to be specified in terms of the modulations of MSRC and MEA they induce together with their motivational/intentional and contextual factors (in addition to specific instructional descriptions of the techniques themselves). In line with this approach, mindfulness in the context of MBAs could be defined as a form of mental training aiming to enhance the MSRC of the mind, particularly targeting the development of attention control and metacognitive awareness of mental phenomena together with cultivation of an attitude of non-reactivity. Through development of the MSCM, mindfulness also creates basic conditions for initial shifts in MEA termed in the MBA context as decentering. Importantly, such shifts in MEA are not to be equated with mindfulness because they also require facilitation by processes and qualities developed through other contemplative practices such as those training in compassion and loving kindness, etc., supporting expansion of motivational/intentional factors, and deepening contemplative insight.

Based on this definition of mindfulness, the primary aim of secular mindfulness-based approaches could be described as training in mindfulness as a meditation technique enhancing introspective awareness and attention together with non-reactivity. Development of further emotional and intentional qualities through mindfulness-based approaches depends on inclusion of other, mindfulness non-specific, teacher qualities, and contemplative practices in the courses, e.g., those supporting implicit and explicit cultivation of kindness, compassion, etc., together with indirect support of motivational/intentional modifications. Core mindfulness training combined with further contemplative practices and self-reflective inquiry may result in initial shifts in MEA such as decentering from mental phenomena. More advanced shifts in MEA most likely need to be facilitated by additional contemplative training, typically within an established traditional contemplative system which can provide targeted training in a progression of practices (including further development of motivational/intentional factors) aimed at facilitating deep contemplative insight.

Interestingly, the definition of mindfulness applied in MBAs is different from some traditional definitions of mindfulness in the Buddhist context. The latter often define mindfulness (Pāli: sati; Sanskrit: smṛti; Tibetan: drenba) as a mental faculty enabling holding (recollection) and sustaining of focus on a meditation object (breath, sacred statue, etc.; e.g., Wallace, 1999a; Dorjee, 2013). Mindfulness is here distinguished from introspective meta-awareness (Pāli: sampajañña; Sanskrit: samprajanya; Tibetan: shéshyin) which enables monitoring of attention focus and noticing distractions (Wallace, 1999a; Dorjee, 2013). In some of these descriptions mindfulness is considered a neutral faculty (Thera, 1998) and in others a positive (virtuous) one when applied in conjunction with other qualities (e.g., Olendzki, 2008). Enhancements in mindfulness are not sufficient for shifts in MEA, these would arise only in combination with further training in emotional qualities of loving kindness, compassion, rejoicing, and equanimity coupled with development of motivational/intentional factors. So in terms of the proposed conceptualizations of contemplative science, mindfulness in this context would be selectively enhancing some aspects of MSRC, mostly attention processes, without significant implications for MEA or involvement of motivational/intentional factors.

Much of previous discussion in the literature on definitions of mindfulness (e.g., Williams and Kabat-Zinn, 2013) was about finding the ‘right’ definition for the term. However, the differences in definitions of mindfulness highlighted here in the context of the proposed framework of contemplative science suggest that rather than striving for ‘the correct and unanimous’ definition of mindfulness, it might be more productive to focus on accurate descriptions of specific contextualized meanings of the term mindfulness. The same principle holds for definitions of other contemplative practice. Accordingly, the associated terminological and conceptual confusions might be greatly minimized, if the similarities and differences of varied definitions of contemplative practices are clearly presented via comprehensive systematic definitions. Within the proposed framework, such definitions should include (1) clear instructional descriptions of how the practice is done; (2) outline of motivational/intentional and contextual factors of the practice; (3) expected pattern of MSRC modulations (including, metacognition, attention, emotion regulation, and conceptual processing); and (4) expected modulations in dereification processes associated with distinct MEA. Such definitions would, for example, make it clear how mindfulness cultivated in MBAs differs from other conceptualizations of mindfulness in the Buddhist context, and also enable comparisons between modulations resulting from different types of mindfulness training and other contemplative practices.

### Contemplative Science in Broader Scientific Context

A clear and uniquely distinctive interdisciplinary methodological approach of contemplative science is also likely to highlight its potential for making a pioneering contribution to psychological and neuroscientific research. Of particular broad interest in this regard might be focused investigation of the interplay between motivational/intentional factors, metacognitive SR, MEA, and well-being outcomes. Such research could provide new insights into psychological and neurocognitive mechanisms underlying mental and physical health with strong implications for prevention and treatment.

In addition, advancements in introspective methodologies, and in their integration with behavioral and neurocognitive assessments, may contribute to a broader methodological shift in psychological and neuroscientific research, possibly providing new insights into some of the most persistently challenging topics including the theory of mind and the nature of conscious experience. Furthermore, the constructs of MSRC and MEA, while specifically targeted in contemplative training and hence essential in contemplative science research, are themselves non-restricted to the discipline and as such of potential relevance, for instance, in developmental, education and consciousness research. In sum, contemplative science defined as an interdisciplinary field of study of the MSRC and MEA modulated by motivational/intentional and contextual factors has a strong potential to enrich psychological and neuroscientific research through its unique theoretical and empirical investigations.

### Recommendations for Future Research in Contemplative Science

The main challenges of meditation research have been recently extensively discussed by Davidson and Kaszniak (2015) and also previously considered by others (e.g., Davidson, 2010; Desbordes and Negi, 2013; Tang et al., 2015). Assessment of first-person experiential aspects has been their central theme with the neurophenomenological approach proposed as a solution. Other persistent methodological challenges include measuring constructs such as mindfulness and the need for more rigor in research designs applied in the field, including more clarity and detail in descriptions of contemplative practices and participant samples (Davidson and Kaszniak, 2015). Less often highlighted, but equally important, is the lack of replication of findings which diminishes the weight and implicational strength of meditation research findings, particularly those from experimental and neuroscientific investigations.

In addition to these essential methodological recommendations, the proposed framework of contemplative science highlights four further recommendations which could contribute to advancement of contemplative science theory and empirical research on meditation: (1) systematic comprehensive definitions of contemplative practices including instructional descriptions, specification of intentional/motivational and contextual factors of the practice, outline of processes of MSRC targeted by the practice and expected impact of the practice on dereification processes and MEA (see section “Contemplative Science in Broader Scientific Context”); (2) development of comprehensive theory specifying MEA and their associations with processes of the MSRC, motivational/intentional and contextual factors; (3) refinement of the neurophenomenology approach and development of further first-person and second-person methods to capture phenomenological experience through self-interpretation or expert-interpretation without temporary delay (see section “Research Methodology of Contemplative Science”) and their systematic inclusion in empirical research on meditation; (4) targeted investigation of same underlying cognitive mental constructs across studies and variety of contemplative practices to enable development of an overarching contemplative science theory integrating findings and guiding further systematic research. As with the methodological suggestions outlined previously (Davidson and Kaszniak, 2015), the main challenge for the field of contemplative science remains in translating such recommendations into actual research practice.

### Limitations of the Proposed Framework of Contemplative Science

The core intention behind the proposed framework is to support systematic investigation of contemplative practices across contemplative traditions. The main limitation of the framework in its current form is that, despite its broad applicability intent, it is mostly based on understanding of the progression of practices in the Buddhist context (with some aspects particularly inspired by the Tibetan Buddhist tradition of Dzogchen) and in secular MBAs. Such approach seems unavoidable at the current stage of research in meditation given that majority of previous studies focused on Buddhist meditation and its secular applications, thus creating a basis for further contemplative research. Future theoretical and empirical research on contemplative practices in non-Buddhist traditions will determine the extent to which the framework is suitable for investigations in contemplative science more broadly as intended. Another limitation of the proposed framework in its current form is its specification in overarching terms which needs to be further refined in future theoretical and empirical research (e.g., involvement of specific attention processes and networks, detailed discussion about metacognitive aspects of contemplative practices, refinement of the progression of MEA, etc.). Some of these limitations will, hopefully, be addressed in further research which this paper aimed to stimulate.

## Conclusion

This paper outlined one possible definition of contemplative science and its subject as a means of addressing some persistent challenges in current research of contemplative practices such as terminological and definitional confusions and theoretical limitations. It has been suggested that contemplative science can be defined as an interdisciplinary study of the MSRC of the mind and MEA with both modulated by motivational/intentional and contextual factors of contemplative practices. Advantages of the proposed definition for guiding further systematic research have been discussed together with possible implications for definitions of mindfulness and other practices. It has been suggested that the outlined framework could make research in contemplative science more inclusive of varied contemplative practices and enable contemplative science to harness more fully its potential in making a unique contribution to psychological, cognitive science, and neuroscientific research. The paper highlighted that meditation research seems to be moving into a more mature scientific phase, where considerations of its key theoretical and definitional issues need to take a central stage to enable its further advancement.

## Author Contributions

The article has been solely authored by DD without contribution of other authors, including initial ideas, their progression, arguments presented in support of the proposal and the models presented in **Figures 1** and **2**.

## Conflict of Interest Statement

The authors declare that the research was conducted in the absence of any commercial or financial relationships that could be construed as a potential conflict of interest.

## Footnotes

1 The term contemplation (and its derivatives) is in this article used as inclusive of meditation, but is considered somewhat broader than meditation since it includes practices (such as different types of prayer or energy movement practices) which would not be described as meditation in some contemplative traditions.

2 Another framework of self-regulation developed by Baumeister and Heatherton (1996) is commonly applied in clinical/health psychology context. This approach has not been considered in recent research on mindfulness/meditation and self-regulation (e.g., Tang et al., 2015), which might be due to difficulty in translating some psychological constructs, such as ‘standards,’ etc., proposed in this theory into cognitive and neurocognitive constructs. The current paper stays within the self-regulation framework typically discussed in meditation research.

3 In the context of meditation research the term ‘intention,’ rather than ‘motivation,’ is more frequently used (e.g., Shapiro et al., 2006). For example Shapiro (1992) suggested developmental progression of intention from self-regulation, through self-exploration to self-liberation as a result of continuous meditation practice. In the framework proposed here, it is suggested that motivational factors need to be included in conjunction with intentional factors, primarily as a means to connect this aspect of contemplative science investigation with existing theories and empirical research on motivation theory in psychology and cognitive science (e.g., similarities and differences between humanistic theories of motivation and motivational factors in the Tibetan Buddhist tradition of Dzogchen are considered in Dorjee, 2013)

4 Attention, particularly attention control, and metacognition are to some extent overlapping processes (see for example Malinowski, 2013). Here they are singled out separately to highlight introspective metacognition as a unique aspect of self-regulation specifically targeted by contemplative practices.

5 In the contemplative context the interpretation of what is considered ‘adaptive’ may strongly depend on the motivational/intentional factors and contemplative context of a practice and in some aspects diverge from standard psychological understanding in terms of situational or goal-directed appropriateness; the notion of well-being conduciveness is often linked to specific understanding of virtue and non-virtue within a contemplative system.

6 In the context of Dzogchen, the purpose of practices enhancing the MSRC of the mind could be described in terms of settling the ‘ordinary mind’ so that more advanced MEA can be recognized. The intentional/motivational and contextual factors can be viewed as essential catalysts in this process.

7 The ICS suggests that implicational meanings are linked to emotional aspects of experience whereas propositional meanings are not – a somewhat controversial claim which is not of essence here.

8 http://cognitive-edge.com
